# Global health: Integrating national laboratory health systems and services in resource-limited settings

**DOI:** 10.4102/ajlm.v1i1.11

**Published:** 2012-06-11

**Authors:** Linda M. Parsons, Akos Somoskovi, Evan Lee, Chinnambedu N. Paramasivan, Miriam Schneidman, Deborah Birx, Giorgio Roscigno, John Nkengasong

**Affiliations:** 1 Global AIDS Program, US Centers for Disease Control and Prevention, Atlanta, USA; 2 Foundation for Innovative New Diagnostics, Geneva, Switzerland; 3 The World Bank, Washington, DC, USA

## Abstract

Laboratory systems worldwide are challenged not only by the need to compete for scarce resources with other sections of national health care programmes, but also with the lack of understanding of the critical role that laboratories play in the accurate diagnosis and monitoring of patients suffering from high-burdens of disease. An effective approach to establishing cost-effective laboratory systems that provide rapid and accurate test results for optimal impact on patient care is to move away from disease-specific programmes and establish integrated laboratory services. An integrated laboratory network provides all primary diagnostic services needed for care and treatment without requiring patients to go to different laboratory facilities for specific tests. Such a network focuses on providing quality-assured basic laboratory testing through the use of common specimen collection, reporting and diagnostic platforms that can be used across diseases. An integrated laboratory system also provides specimen transport to specialised laboratories and an environment conducive to the introduction and use of new and more complex technologies that would benefit the patient population and public health systems as a whole. As such, this article described various strategies for, and practical examples of, the successful integration of laboratory services.

## Introduction

Human immunodeficiency virus (HIV) and/or AIDS, tuberculosis (TB) and malaria remain major threats that undermine the health of global populations and account for approximately five million deaths every year.^1^ A lack of adequate laboratory capacity in resource-poor, high-burden countries presents a significant barrier in providing appropriate diagnosis, care and treatment to patients infected with these and other emerging pathogens.^1,2,3^ Modernisation and quality improvement of laboratory services would greatly improve the control of these diseases and support other initiatives.^4^ Decades of underinvestment in national laboratory programmes have resulted in deficiencies in the infrastructure, equipment and human resources that are necessary for effective, quality-assured laboratory services.^5^ Increases in international funding have been unprecedented in recent years,^6,7^ but programmes for major diseases are still organised vertically as silos, including the establishment of similar testing technologies for different diseases. This has led to wasted resources because of duplications of equipment and training and inefficient use of human capacity. During this time of increased awareness and programmatic scale-up, it is imperative to establish integrated national laboratory systems to optimise quality, efficient and cost-effective testing.

## What does integration of laboratory systems and services mean?

The demand for laboratory services to meet the diagnostic and treatment needs for HIV has helped drive investment in new and renovated laboratories to provide comprehensive laboratory diagnostic services for monitoring patients on anti-retroviral therapy with CD4, chemistry, haematology, molecular tests such as measuring plasma Ribonucleic acid (RNA) levels, testing of HIV-1 drug resistance mutations and testing for opportunistic infections. However, this expansion and investment in laboratory capacity should be optimised to serve the needs of other diseases of public health importance, provide general diagnostic services to support clinical care and support other public health priorities such as re-emerging infectious diseases and chronic diseases.

An integrated laboratory network can be defined as one that has the ability to provide all primary diagnostic services needed for the care and treatment of patients without requiring them to go to different laboratories for specific tests. To meet these requirements the network should, (1) be focused on providing quality-assured basic laboratory testing, (2) use common specimen collection, timely reporting and diagnostic platforms that can be used across diseases within the same facility and (3) increase capacity for introducing and using new and more complex technologies.

## Advantages of integrated laboratory systems

Integrated systems have several advantages. Firstly, and most importantly, they can support timely and comprehensive care and treatment without the costs and delays associated with referral to specialised facilities for particular diagnostic exams. An integrated system can benefit patients and clinical staff at hospitals located in urban centres, as well as patients served at the community level in remote areas. Secondly, new diagnostic platforms, such as microscopes with dual light and fluorescent capacity and molecular diagnostic equipment, have the potential to perform assays for more than one disease. An integrated laboratory approach will ensure that these tools can be used optimally by cross-trained technical staff. Thirdly, integrating diagnostic services for different diseases within the same facility helps avoid duplication of investments in infrastructure and laboratory supporting systems, such as specimen transport, supply chain management and information systems. Lastly, an integrated approach to training can help ensure standardisation of core laboratory issues, such as quality assurance and standard operating procedures, as well as ensure the more efficient delivery of training. The examples below show how these approaches are both practical and successful.

## Disease-specific programmes impede laboratory services integration

Laboratory systems in resource-poor countries are challenged by, (1) dilapidated infrastructures, (2) lack of funding for developing and implementing national policies, strategic planning, and quality management systems, (3) unlinked referral and reporting services and (4) inadequate human resources, including lack of organised in-service training and long-term career pathways. The difficulty of dealing with all these challenges separately by each programme should provide sufficient reason to encourage integration of services. However, overcoming the traditional vertical approach in which higher level public health laboratories have been established to provide support for disease-specific ministerial directorates and programmes has proven difficult. Furthermore, an unintentional negative effect of the disease-specific approach to laboratory strengthening has been the neglect of the core public health laboratory functions. Most often, integrated laboratory services are usually found only at the district, sub-district and primary health centre levels. However, establishment of parallel laboratories within a national health care system is neither cost-effective nor conducive to efficient and coordinated patient care.

## Strategies for integrating laboratory systems and services

Integration of laboratory systems and services at different levels of the health system will ensure optimisation of the investments in laboratory-system strengthening by Ministries of Health, as well as those of international donors, such as the Global Fund to Fight AIDS, TB, and malaria, the World Bank, the US President’s Emergency Plan for AIDS Relief (PEPFAR), and others. In the past, donor funding mechanisms have contributed to the fragmentation of laboratory capacities because they usually have been earmarked for disease specific efforts; although, recently, there is a trend towards greater flexibility. Various strategies for integration are outlined in the subsections below.

### Policy and strategic planning

National laboratory systems (ideally composed of a tiered network of diagnostic laboratories and a National Public Health Reference Laboratory) must be capable of providing accurate, timely and cost-effective testing that is in line with each country’s programmatic goals and available clinical interventions. It is necessary to define at which level of a national laboratory network certain services or diagnostic platforms should be performed. These decisions should be based on testing complexity, throughput, specimen referral requirements and the needs of the public health programme and patient population being served. Laboratories are complex systems that include components such as personnel, equipment, supplies and infrastructure, as well as support systems for information management, purchasing and inventory, and systems for evaluation and continuous improvement, such as internal and external quality assessment and occurrence management. Developing and implementing a national laboratory policy and strategic plan that ensures integrated capacity at each level of the network can enable countries to work with partners and donors in defining specific objectives, setting standards and allocating appropriate funding and personnel for sustainable laboratory services.^8^

### Standardising testing and equipment and coordinating with partners

Laboratory infrastructure, test menus, technology, platforms and commodities should be standardised in each country to avoid duplication, as agreed upon by programme and laboratory representatives from 13 resource-poor countries in the Maputo Declaration of 2008.^9^ This approach requires strong leadership and coordination by the local ministries, along with partner and donor compliance, and includes many benefits, such as reduced procurement costs for commodities, easier implementation of quality assurance programmes and integration of multi-focused testing that uses shared equipment. For example, partners could work together in developing specialised facilities that would accommodate instruments for molecular diagnostics of TB, HIV and other diseases, rather than building separate labs dedicated to only one disease. Training, equipment maintenance and quality management systems could be standardised to allow technicians to work using similar techniques across diseases. Joint coordination amongst partners at the onset of a laboratory-related project could enable each partner to play a preferred role in areas where they have a relative advantage or expertise.

### Joint coordination with clinicians

Strengthening of national laboratory systems depends on close partnerships – beyond the laboratory facility itself – with technical and clinical professionals, healthcare managers at the community, regional and national levels, and public health programmes. Clinicians should support, facilitate and demand high-quality and responsive laboratory support for appropriate patient care. Clinicians should also be involved in ensuring that testing algorithms are cost-effective, are based on sound evidence and have a relevant impact on clinical decision-making and patient-important outcomes.^10^

### Implementation

**FIGURE 1:**
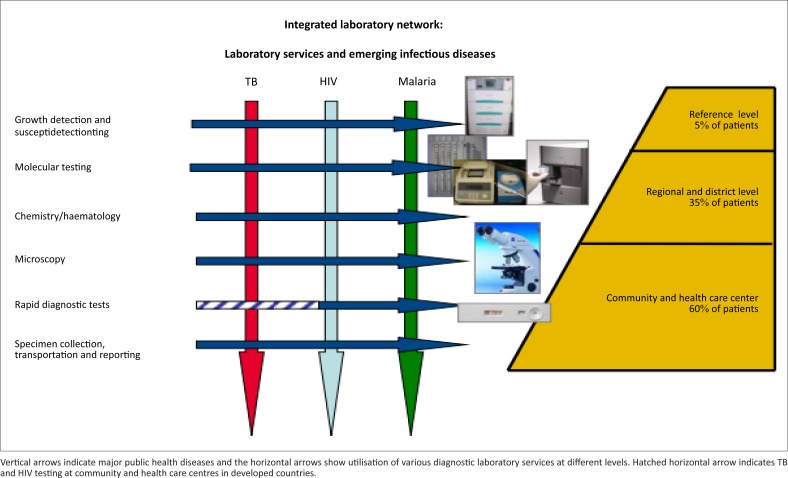
The framework of integrated laboratory services that addresses levels of a tiered laboratory network in developing countries.

There are two basic aspects to implementing integrated services within laboratories. The first is focused on provision of adequate services for patients presenting with particular clinical symptoms indicative of major infectious diseases. For example, an integrated laboratory should have the capacity to adequately monitor HIV-positive individuals for TB, malaria, or other opportunistic infections, or to provide rapid molecular testing for multi-drugresistant TB (MDR TB) in HIV and TB co-infected patients in order to improve infection control and treatment outcomes (Figure 1). Conversely, this laboratory should also be able to screen specimens from TB and malaria patients for HIV and provide other routine monitoring (clinical chemistry and haematology before and during treatment for HIV, TB, and malaria) when required.

The second aspect focuses on the establishment of integrated diagnostic platforms or instruments that can be used for a variety of tests. The simplest example can be an integrated system for collecting and transporting specimens for TB and HIV testing or monitoring. A further step can be the integration of microscopy testing for TB, malaria and other parasitic pathogens. The most promising approach for cross-cutting disease testing is offered by the molecular platform. Polymerase chain reaction (PCR) molecular testing can be used to detect rapidly and identify a wide range of viral and bacterial pathogens, including those that cause endemic diseases such as HIV, TB and malaria, and emerging infectious diseases such as new strains of influenza.

## Practical examples of integrated laboratory capacity in the field

### Integration of TB laboratory capacity in HIV laboratories

In 2006, a state of the art bio-safety level (BSL)-2 central laboratory was established at the Ethiopian Health and Nutrition Research Institute (EHNRI) in Addis Ababa through PEPFAR funding to support the laboratory diagnosis of HIV patients. Since then, technical support has been provided by international partners to help establish testing and training capacity for HIV serology and external quality assessment (EQA), HIV incidence, CD4 counts, biochemistry, haematology and molecular testing for viral load and early infant diagnosis (EID). Beginning in 2008, support was provided through the Foundation for Innovative New Diagnostics (FIND) to create two BSL-3 TB testing laboratories in Addis Ababa: the first at the HIV laboratory at EHNRI, to host the National Reference Laboratory for Tuberculosis (NRLT), and the second at St. Peter’s Hospital, a central hospital responsible for the care of TB and HIV co-infected patients experiencing TB treatment failure or relapse. The testing capacity of the two laboratories now includes liquid and solid growth detection and drug susceptibility testing for TB, lateral-flow immuno-assay for identification of TB and rapid detection of MDR TB by the molecular line probe assay (LPA). The newly renovated molecular unit at St. Peter’s will also be used to restart HIV viral load testing at the hospital, a function that had been stopped in the past because of problems related to inadequate infrastructure for molecular testing. In addition, molecular LPA testing for MDR TB will soon be introduced in four Ethiopian regional laboratories that are currently performing HIV DNA PCR for EID.

In Nigeria, a multifaceted, integrated, tiered laboratory programme has been established by the Institute of Human Virology at the University of Maryland to support a PEPFAR-funded scale-up of AIDS care and treatment in 26 states.^11^ Services provided by the laboratory network include HIV rapid tests, adult CD4 counts, paediatric CD4 percentages, haematology, blood chemistries, syphilis serology, cryptococcal antigen test, TB smear microscopy, culture, drug susceptibility testing (DST), and molecular LPA. Use of appropriate technology at all service levels and a robust quality assurance programme provide high quality integrated laboratory services.

### Integration of HIV testing in a TB laboratory

The National TB Reference Laboratory (NTRL) located at the Queen Elizabeth II Hospital in Maseru, Lesotho, was renovated by FIND and other partners to provide capacity for TB culture and molecular diagnostics. Following strengthening of TB smear microscopy testing through training and the establishment of a quality assurance programme, the NTRL was first renovated to create a BSL-3 facility to implement TB culture, DST and rapid immunoassay-based identification of TB, with EQA provided from South Africa.^12^ Then TB LPA and HIV DNA PCR were implemented in an adjacent, newly constructed clean-room facility, resulting in the establishment of integrated TB and HIV testing in a single facility. By using an integrated approach, implementation of novel molecular TB diagnostics paved the way to introduce HIV molecular testing capacity in the country.

### Avian influenza laboratory support in HIV laboratories

Molecular HIV laboratories in Africa have been called on to respond rapidly to laboratory surveillance for outbreaks of avian influenza. During the 2005 outbreak of avian influenza, the PEPFAR-supported Global AIDS Program laboratory in Entebbe, Uganda, provided training for about 60 laboratory experts on the use of PCR diagnosis for avian influenza. Also in 2006, the PEPFAR-supported laboratory at Asokoro Hospital in Abuja, Nigeria, was instrumental in providing diagnostic support for an investigation of an avian influenza outbreak in Nigeria.^13^ Planning is ongoing for the provision of support for localised outbreaks of H1N1 (swine) influenza.

## Human resources training

The African Centre for Integrated Laboratory Training (ACILT) was established in 2008 to develop and offer hands-on training courses for front-line laboratory staff. ACILT’s vision is to provide for a healthier Africa through quality laboratory practices to combat major infectious diseases. Hosted by the South African National Institute for Communicable Diseases and the National Health Laboratory Service, ACILT has a governance board consisting of experts from collaborating international institutions.

Before establishing ACILT, needs assessments were performed in 10 resource-poor African countries to determine which topics would be most important for building capacity amongst laboratory personnel. Courses were then developed collaboratively by ACILT and international partners including the US Centers for Disease Control and Prevention (CDC), FIND and the World Health Organization to ensure standardisation. As of early August 2011, 720 participants from 29 countries had participated in more than 20 hands-on training courses covering: HIV DNA PCR for EID, HIV incidence assay, TB culture and identification, EQA for HIV rapid testing, national laboratory strategic planning, biosafety, laboratory management, and laboratory accreditation.

ACILT satellite courses are offered at sites outside of South Africa. A two-week course on TB culture and drug susceptibility testing was recently offered to South-East Asian participants in Bangkok and one-week courses on the molecular LPA testing for MDR TB have been hosted by the new laboratory facility at EHNRI, at the National TB Reference Laboratory of Lesotho, and at the Institut Pasteur in Abidjan, Côte d’Ivoire. ACILT TB culture and molecular LPA course materials have also been translated into French and Portuguese through a partnership with the American Society for Microbiology.

## Recommendations and conclusion

An integrated network of laboratories is indispensable for providing national support for global health initiatives for HIV, TB, malaria and other public health priorities. However, an integrated national laboratory network should be able to provide all needed primary diagnostic services for patients at each level of service without requiring patients to go to different facilities for specific tests. Such laboratory services might be delivered either directly at defined levels of the network or indirectly by using an integrated specimen collection, referral and reporting system. The extent of integration at different levels of the laboratory system should always be based on the complexity, throughput and specimen referral requirements of the particular diagnostic platforms. Last but not least, an integrated system allows increased capacity and preparedness for implementation of new technologies to improve the quality of current diagnostics and to address newly emerging public health concerns. This article has presented examples to demonstrate how some resource-limited countries have benefited from an integrated approach for clinical laboratory strengthening. These strategies could be replicated more broadly.
